# Broadband frequency-reconfigurable metamaterial absorber using switchable ground plane

**DOI:** 10.1038/s41598-018-27609-5

**Published:** 2018-06-15

**Authors:** Heijun Jeong, Sungjoon Lim

**Affiliations:** School of Electrical and Electronic Engineering, Chung-Ang University, Heukseok-Dong, Dongjak-Gu 156-756, Republic of Korea

## Abstract

In this study, we propose a broadband frequency-reconfigurable metamaterial absorber using a novel switchable ground plane (SGP). A double resistive square-ring resonator is introduced for broadband absorption. The distance between the top resonator pattern and the ground plane determines the resonant frequency; the proposed SGP is thus capable of switching the absorption frequency band. The SGP can be either ground or reactive, by switching the PIN diodes on and off, respectively. The SGP is placed as the middle layer, between the top pattern and the bottom ground plane. In the low frequency band, the SGP becomes reactive and the bottom ground plane works as the ground plane of the absorber. In the high frequency band, the SGP works as the ground plane and the bottom ground plane does not affect the absorber. The proposed idea is demonstrated via full-wave simulations and measurements. The absorption of the fabricated sample with 27 × 27 unit cells is measured under normal incidence. When the PIN diodes of the SGP are turned on, an absorption higher than 90% is achieved between 3.5–11 GHz. When the PIN diodes of the SGP are turned off, an absorption higher than 90% is achieved between 1.7–5.2 GHz.

## Introduction

Metamaterials are artificial structures that have an infinite array of periodic structures^1^. We can control the permittivity and permeability of a material by making use of metamaterials. The properties of metamaterials can be applied to electromagnetic (EM) absorber technology; an EM absorber is a device that consumes EM energy to inhibit the formation of reflected waves. EM absorbers are used in many fields and applications, such as stealth technologies^2,3^, electromagnetic interface (EMI) and electromagnetic compatibility (EMC) solutions^4^, sound wave attenuation^5^, human body applications^6^, and super lenses^7,8^. EM absorbers can be divided into material-based absorbers, such as wedge-tapered absorbers^9–14^ and Jaumann absorbers^15^, and metamaterial-based absorbers^16^. Wedge-tapered absorbers that use ferrite materials have an excellent absorption capacity, but they are bulky and expensive. To overcome these drawbacks, Jaumann absorbers are composed of a dielectric material with quarter-wavelength (λ/4) thickness and a resistive sheet to make them thinner than wedge-tapered absorbers. However, they are still bulky. On the other hand, metamaterial absorbers absorb EM energy through a resonance structure and exhibit an absorption rate close to 100%, even with a very thin structure. Moreover, they are cost-effective and easy to fabricate.

Despite these advantages of metamaterial absorbers, the use of a resonant frequency is disadvantageous because a high absorption rate is achieved only near the resonant frequency, making them effective only in a narrow band. For instance, radar spectra are not a single narrow frequency band, as various frequencies are used for radar applications. Therefore, broadband absorbers are required for practical applications. Due to this demand, various broadband frequency, frequency-tunable metamaterial absorbers have been actively studied in order to increase their absorption frequency bands. Out of the various methods that have been studied, the first one involves using multiple resonances^17–20^. The second method uses a resistive pattern^21–23^. A broader bandwidth can be obtained by designing the resonant pattern of the absorber using lossy materials. The third method consists in using lumped elements, such as chip resistors^24–27^. Not only are lumped elements easy to incorporate in printed-circuit-board (PCB) processes, but their inclusion also provides an excellent broadband performance.

Moreover, in order to achieve broadband absorption, studies have not only been carried out on broadband metamaterial absorbers, but also on frequency-tunable metamaterial absorbers. There are various methods for tuning the frequency of metamaterial absorbers. For example, frequency-tunable characteristics can be achieved by using electronic devices, such as PIN diodes^28–30^, varactor diodes^31^, or micro-electromechanical systems (MEMSs)^32–34^. Frequency-tunable methods using electronic devices are widely used for frequency variation. Despite of their disadvantages, such as requiring a bias line and the expensive cost of their devices, they are still capable of immediate frequency tuning and offer an excellent frequency-tuning performance. Furthermore, instantaneous frequency tuning is essential for detecting radar signals that reveal enemies at high speeds; hence, we propose a frequency-tunable absorber using such electronically active devices.

In this study, we propose a broadband frequency-reconfigurable metamaterial absorber using a novel switchable ground plane (SGP). A double resistive square-ring resonator is introduced for broadband absorption. The distance between the top resonator pattern and the ground plane determines the resonant frequency; therefore, the SGP is used to switch the absorption frequency band. The SGP can be either ground or reactive by switching the PIN diodes on and off, respectively. The SGP is placed as the middle layer between the top pattern and the bottom ground plane. In the low frequency band, the SGP becomes reactive and the bottom ground plane works as the ground plane of the absorber. In the high frequency band, the SGP works as the ground plane and the bottom ground plane does not affect the absorber. In this work, the proposed absorber was successfully demonstrated via simulations and measurements. The proposed idea will be further explained in the following sections.

## Design and Simulation

**Figure 1:**
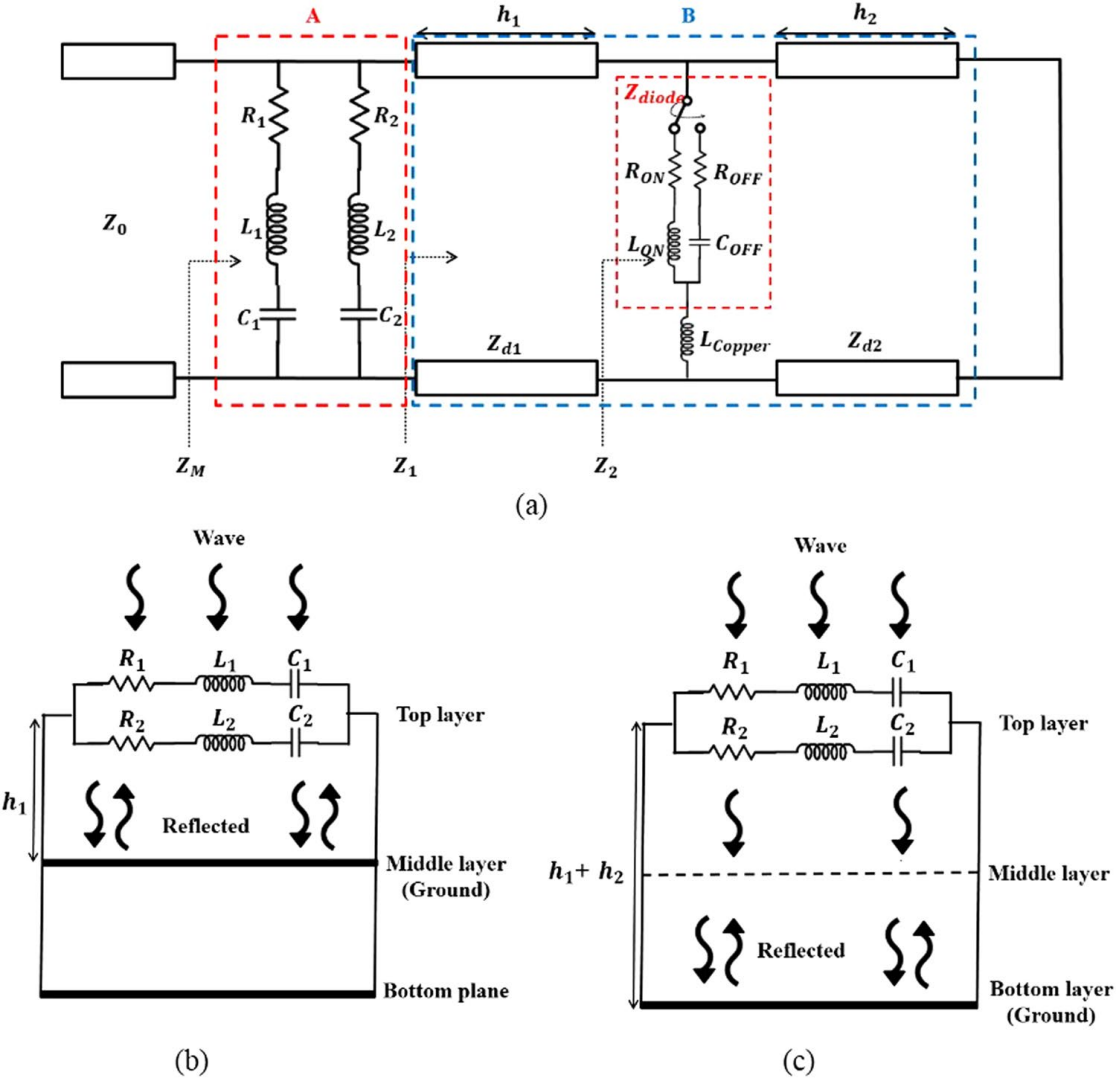
Operating principle of the proposed frequency-reconfigurable absorber using an SGP: (**a**) equivalent circuit model of the proposed absorber; (**b**) the proposed absorber when the SGP switches to the ON state and (**c**) when the SGP switches to the OFF state.

In this study, because the bottom surface is completely covered with the ground plane, the reflection coefficient should be minimized in order to obtain a high absorption. In order to minimize the reflection coefficient, we matched the metamaterial impedance (Z_M_) with the free space impedance (Z_0_). Figure 1 shows the operating principle of the proposed frequency-reconfigurable absorber according to the SGP state. In Fig. 1(a), section A shows an equivalent circuit of the top conductive patterns. The top layers of the two conductive patterns are represented by R_1_, C_1_, and L_1_; and R_2_, C_2_, and L_2_, respectively. Section B shows an equivalent circuit of the middle SGP. The substrate thicknesses are represented by h_1_ and h_2_, and the characteristic impedance of substrates are represented by Z_d1_ and Z_d2_. Furthermore, the equivalent circuits of the PIN diode at the ON and OFF states (Z_diode_) are represented by R_ON_ and L_ON_ and R_OFF_ and C_OFF_, respectively. The pattern of the middle layer is represented by L_copper_. The input impedance (Z_M_) is given by the following equations:

1 $${{\rm{Z}}}_{M}=({{\rm{R}}}_{{\rm{1}}}+{\rm{j}}\omega {{\rm{L}}}_{{\rm{1}}}+\frac{{\rm{1}}}{{\rm{j}}\omega {{\rm{C}}}_{{\rm{1}}}})||({{\rm{R}}}_{{\rm{2}}}+{\rm{j}}\omega {{\rm{L}}}_{{\rm{2}}}+\frac{{\rm{1}}}{{\rm{j}}\omega {{\rm{C}}}_{{\rm{2}}}})||{Z}_{{\rm{1}}}$$

2 $${{\rm{Z}}}_{{\rm{1}}}={Z}_{d1}\frac{{Z}_{2}+{{\rm{jZ}}}_{{\rm{d1}}}\,\tan \,{\beta }_{{\rm{1}}}{{\rm{h}}}_{{\rm{1}}}}{{{\rm{Z}}}_{{\rm{d1}}}+{\rm{j}}{Z}_{{\rm{2}}}\,\tan \,{\beta }_{{\rm{1}}}{{\rm{h}}}_{{\rm{1}}}}$$

3 $${{\rm{Z}}}_{{\rm{2}}}={(Z}_{{\rm{diode}}}+{\rm{j}}\omega {{\rm{L}}}_{{\rm{copper}}})||j{Z}_{{\rm{d2}}}\,\tan \,{\beta }_{{\rm{2}}}{{\rm{h}}}_{{\rm{2}}}$$

4 $${{\rm{Z}}}_{{\rm{diode}}}={({\rm{R}}}_{{\rm{ON}}}+{{\rm{L}}}_{{\rm{ON}}})\,{{\rm{or}}{\rm{Z}}}_{{\rm{diode}}}={({\rm{R}}}_{{\rm{OFF}}}+{{\rm{C}}}_{{\rm{OFF}}}),$$

where β_1_ and β_2_ are the phase constants of the top substrate (h_1_) and the bottom substrate (h_2_), respectively.

The frequency tuning mechanism of the proposed absorber can be explained via the SGP ON/OFF states, as shown in Fig. 1(b,c). When the SGP is in the ON state, the PIN diode of the middle layer operates as a resistor and an inductor (R and L), and thus it operates as if it was shorted. As a result, the middle layer works as a ground plane and is only affected by h_1_. When the SGP is in the OFF state, the PIN diode of the middle layer operates as a resistor and a capacitor (R and C), and thus it operates as if it were open. Therefore, the middle layer operates reactively and its resonant frequency is affected by h_1_ and h_2_.

**Figure 2:**
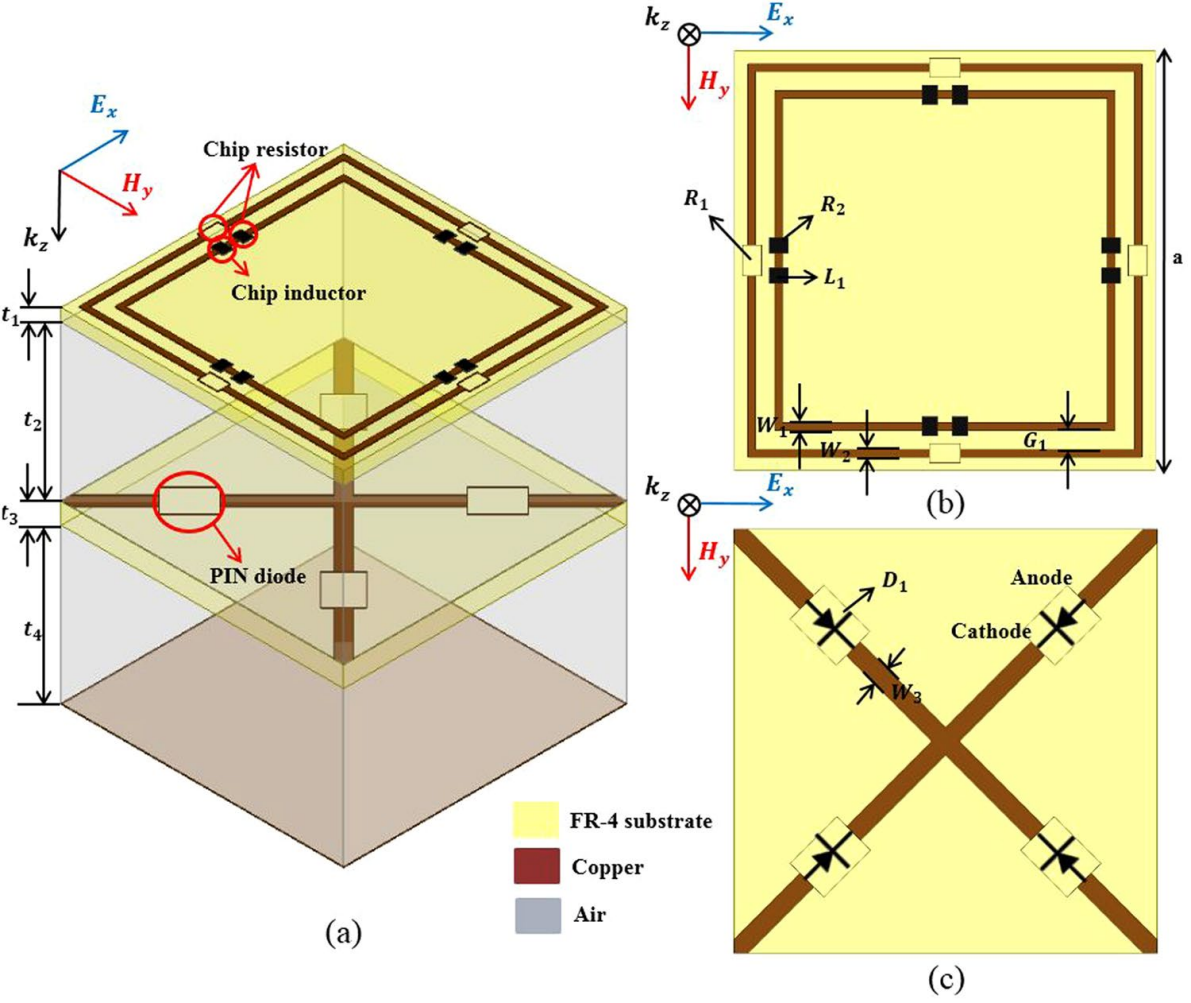
Geometry of the proposed absorber unit cell. (**a**) Perspective view of a unit cell, (**b**) top view of the top layer of a unit cell, and (**c**) top view of the bottom layer of a unit cell.

**Figure 3:**
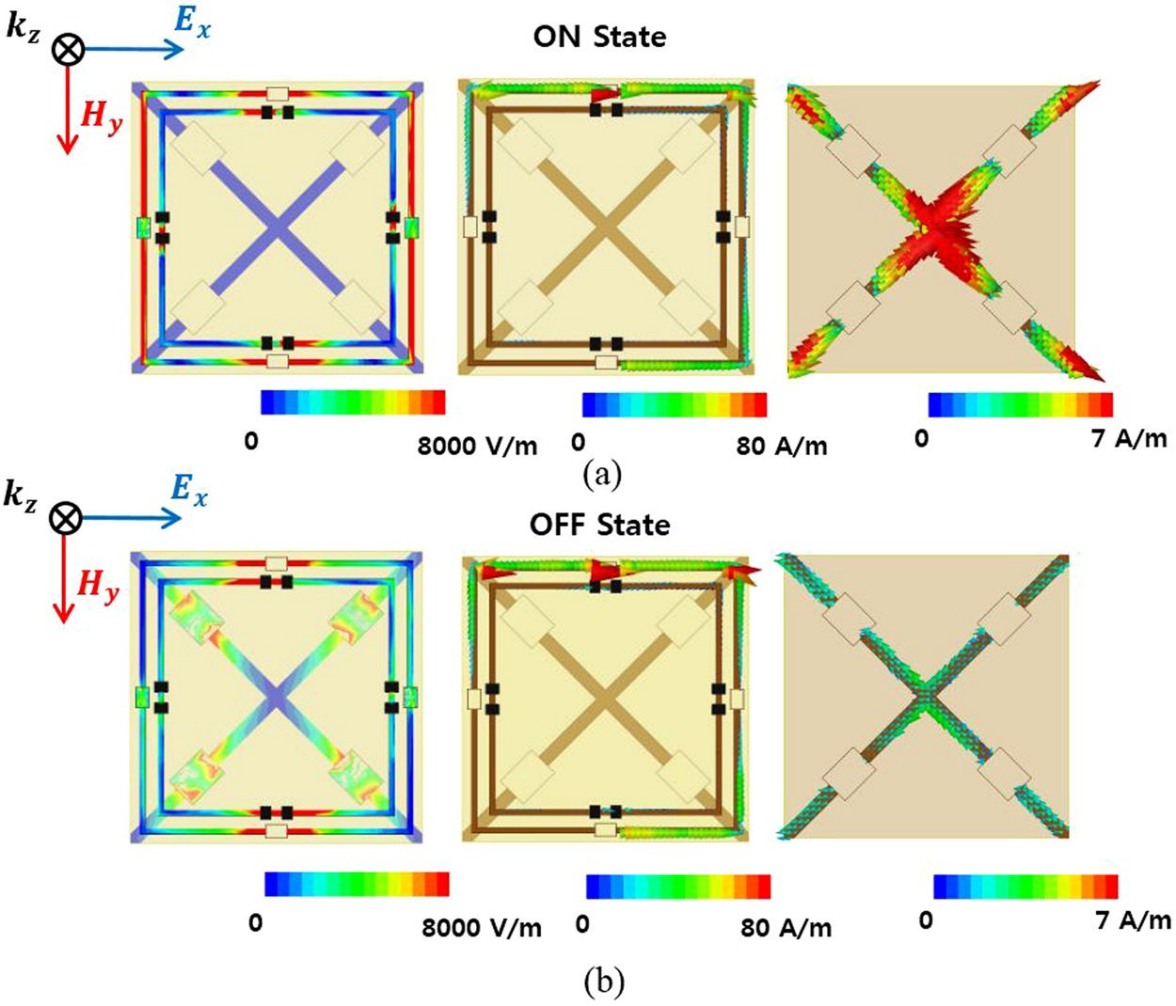
E-field distribution and vector current density of the proposed unit cell according to the state of the SGP. (**a**) ON state and (**b**) OFF state.

Figure 2 shows the geometry of the proposed absorber unit cell, which is introduced in order to physically realize the equivalent circuit presented in Fig. 1(a). To perform our analysis, we used ANSYS high frequency structure simulator (HFSS). As shown in Fig. 2(a), the proposed absorber consists of two fire-retardant or flame-resistant 4 (FR4) substrates with two air layers. The top and middle FR4 substrates (with a dielectric constant ɛ_r_ = 4.4 and a loss tangent of 0.02) have thicknesses t_1_ = 0.5 mm and t_3_ = 0.8 mm, respectively. The thicknesses of the both top (t_2_) and bottom (t_4_) air layers are 6 mm. The top FR4 layer consists of two square copper rings for increasing the bandwidth, as illustrated in Fig. 2(b). The unit cell size is a = 11 mm, and the width of the two rings is W_1_ = W_2_ = 0.2 mm. The outer ring has a chip resistor R_1_ = 100 Ω to increase the bandwidth. Between each ring is a gap (G_1_ = 0.5 mm), and the inner ring also has a chip resistor (R_2_ = 290 Ω) to increase the bandwidth. To make the resonant frequency even lower, we proposed the inclusion of a chip inductor (L_1_ = 47 nH), because only the pattern is limited in this way. The middle FR4 layer consists of the SGP, in which we used four SMP1340-079LF PIN diodes for switching between the ON/OFF states. When the PIN diode switches ON/OFF, the SGP becomes open/shorted, respectively. When the PIN diode is in the ON state, the SGP pattern functions as ground because the PIN diode acts as a short circuit. As a result, the resonant frequency becomes higher as the guided wavelength (λ_g_) becomes smaller. When the PIN diode is in the OFF state, the SGP pattern functions reactively because the PIN diode acts as an open circuit. As a result, the resonant frequency becomes lower as λ_g_ becomes larger. Figure 3 shows the magnitude of the electric field distribution and vector current density of the proposed unit cell. When the SGP is in the ON state, the electric field is distributed over the top conductive patterns, as shown in Fig. 3(a). When the SGP is in the OFF state, as shown in Fig. 3(b), the electric field is distributed between both the top conductive patterns and the middle layer. The vector current density is shown in Fig. 3(a) and, similarly, the current flows in the top and middle layers when the SGP is in the ON state. When the SGP is in the OFF state, a strong current flows in the top conductive pattern.

**Figure 4:**
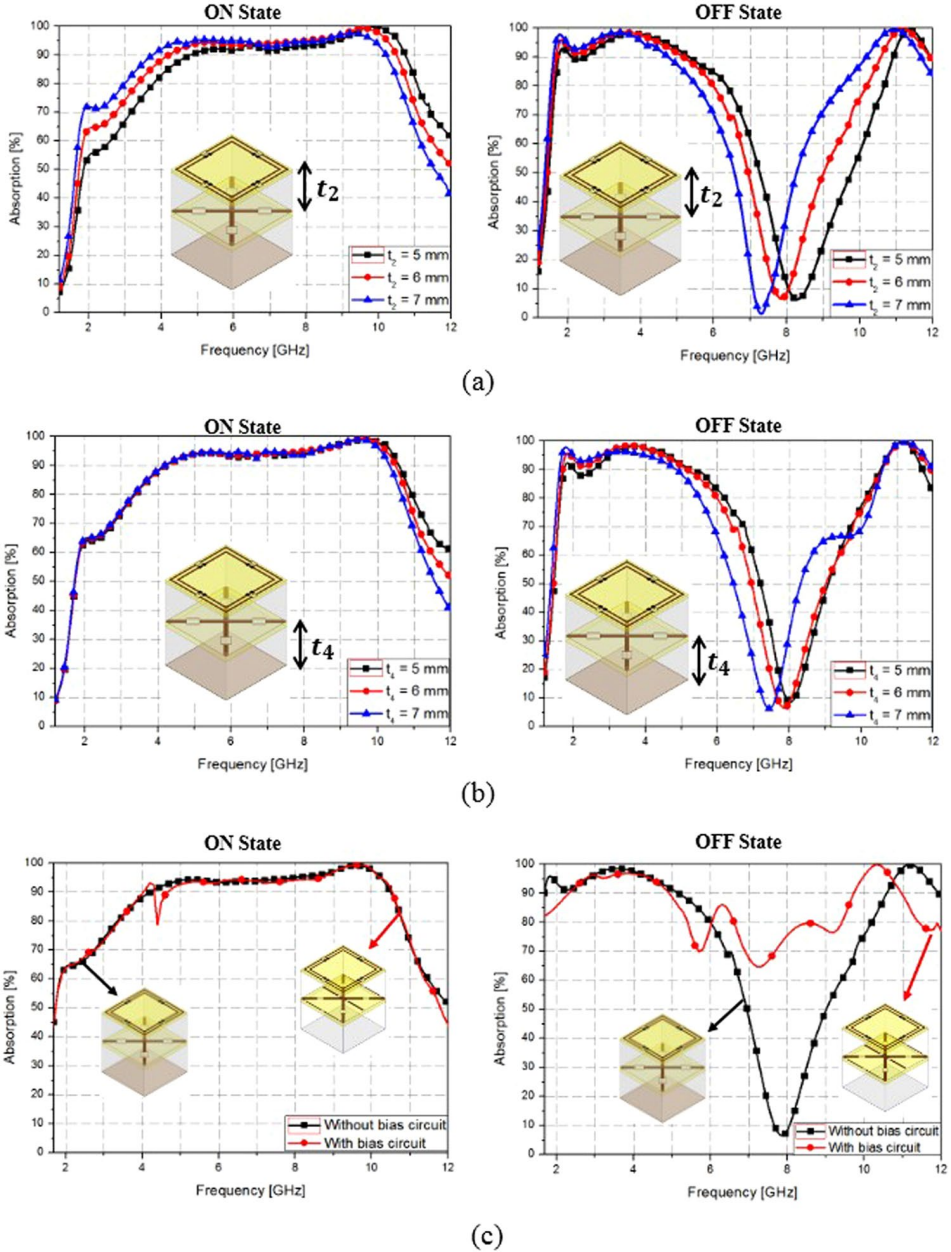
Variations of the absorption bandwidth for different unit cell geometries with the SGP in the ON and OFF states: (**a**) when the thickness of the upper air layer (t_2_) is increased from 5 mm to 7 mm, (**b**) when the thickness of the bottom air layer (t_4_) is increased from 5 mm to 7 mm, and (**c**) without and with the bias circuit for the SGP ON and OFF states.

Figure 4 shows the variations in absorption bandwidth for different unit cell geometries with the SGP in the ON and OFF states. As shown in Fig. 4(a), when the thickness of the upper air layer (t_2_) is increased from 5 mm to 7 mm, it can be seen that the absorption frequency changes in both the ON and OFF states of the SGP. On the other hand, when the thickness of the bottom air layer (t_4_) is increased from 5 mm to 7 mm, it can be seen that there are negligible changes in the absorption frequency when the SGP is in the ON state; the absorption frequency changes only when the SGP is in the OFF state. In order to activate the PIN diodes, a DC bias circuit must be added to the SGP unit cell. Figure 4(c) shows the variations in absorption bandwidth without and with the bias circuit for the ON and OFF states of the SGP. When the SGP without the bias circuit is in the ON state, a 90% absorption bandwidth is observed between 4.21–10.46 GHz. When the SGP with the bias circuit is in the ON state, a 90% absorption bandwidth is observed between 4.1–10.6 GHz. The absorption bandwidth undergoes negligible changes when the SGP is in the ON state. When the SGP without the bias circuit is in the OFF state, a 90% absorption bandwidth is observed between 1.7–5.13 GHz. After including the bias circuit, the 90% absorption bandwidth slightly decreases to 2.3–5 GHz; Absorptivity increases from 5 to 10 GHz, which is outside of the bandwidth. Nevertheless, the proposed metamaterial absorber can switch the absorption band while keeping a wide bandwidth.

## Fabrication and Measurement Results

**Figure 5:**
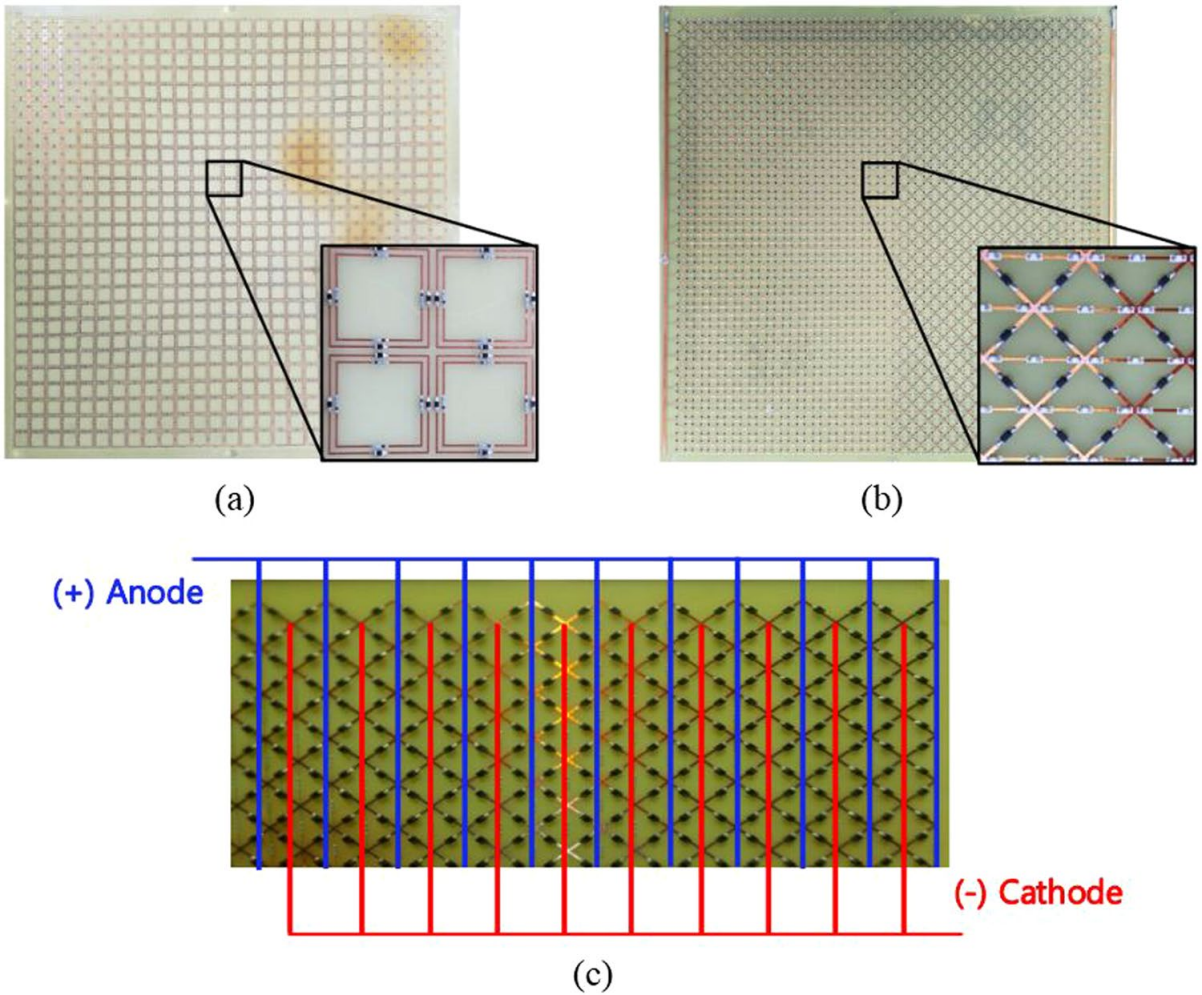
Picture of the fabricated absorber prototype: (**a**) top layer, (**b**) middle layer, and (**c**) DC bias line.

To assess the performance of the proposed absorber, we fabricated a prototype and its DC bias line. Figure 5 shows pictures of the fabricated prototype, including the DC bias lines for the diodes. The overall size of the fabricated prototype was 297 × 297 mm^2^, which consisted of 27 × 27 unit cells. The top layer consisted of a conductive pattern and chip components. The chip resistors used for the outer ring had a resistance of 100 Ω and a size of 1.0 × 0.5 mm^2^ (1005 metric code), and those used for the inner ring had a resistance of 290 Ω and a size of 0.6 × 0.3 mm^2^ (0603 metric code). The inner pattern also had 47-nH chip inductors with a size of 0.6 × 0.3 mm^2^ (0603 metric code) mounted on it. The chip components were mounted via a surface mount technology (SMT) process. The middle layer also consisted of a conductive pattern, and its electric components were mounted via an SMT process. In order to achieve frequency switching, we used an SMP1340-079LF PIN diode. The PIN diode can operate in a frequency range from 10 MHz to more than 10 GHz. In addition, we configured the DC bias line to control the PIN diodes. To prevent radio frequency (RF) signals from interfering at the DC bias line, we used chip inductors for the RF choke. Hence, three chip inductors with a size of 1.0 × 0.5 mm^2^ (1005 metric code) and with inductance and self-resonance frequency (SRF) values of 1 nH and 10 GHz, 5 nH and 7 GHz, and 10 nH and 5.2 GHz, respectively, were mounted.

**Figure 6:**
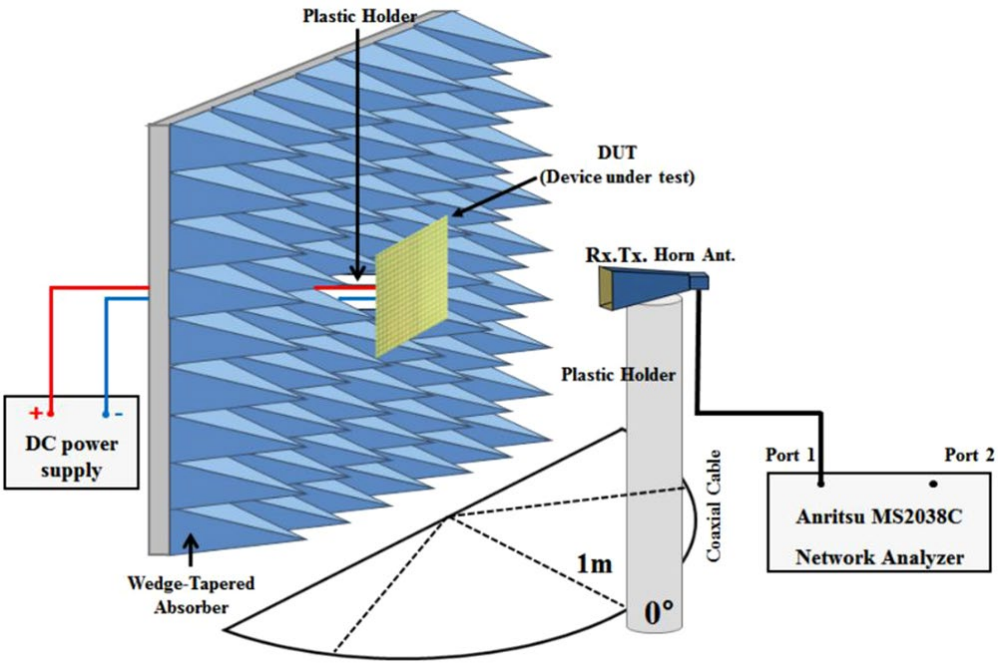
Absorption measurement set-up for normal incidence.

Figure 6 shows the absorption measurement setup for normal incidence in free space. We used a time-gating method using the Anritsu MS2038 vector network analyser (VNA) to measure the signal reflected only from the prototype sample. A wedge-tapered absorber was placed around the prototype sample to prevent scattering from undesired objects. To satisfy the far-field condition, the distance between the sample and the horn antenna was 1 m and a separate DC power supply device was used to activate the PIN diodes.

**Figure 7:**
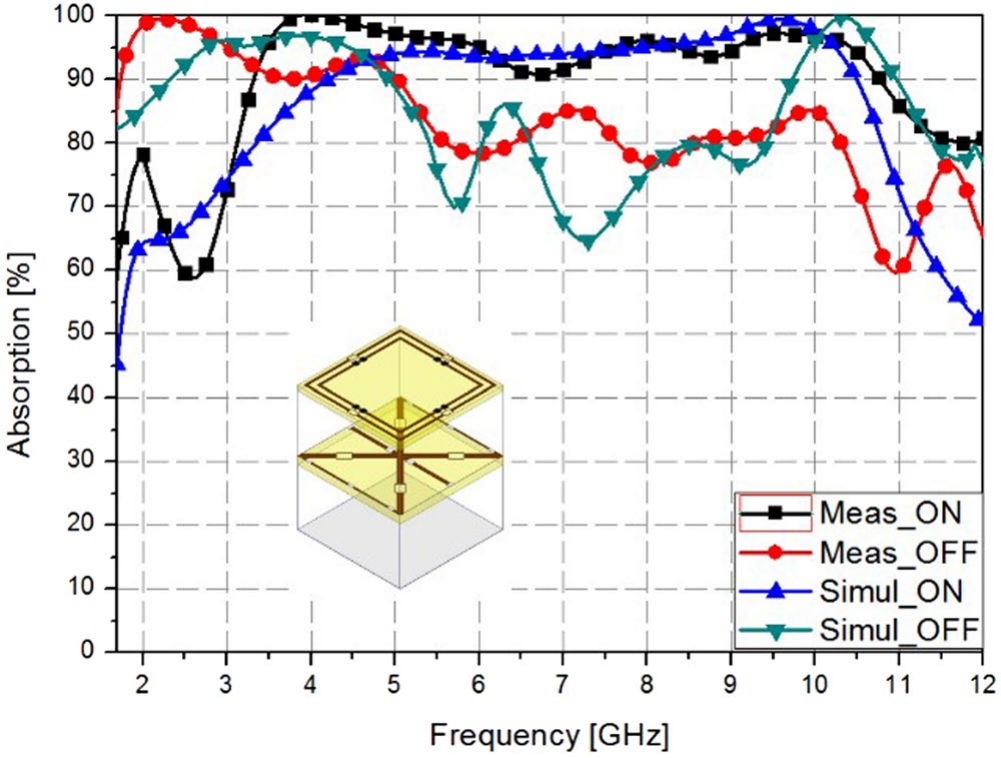
Simulated and measured absorption rates according to the state of the SGP (ON/OFF).

Figure 7 shows the simulated and measured absorption rates according to the state of the SGP (ON/OFF). When the SGP was in the ON state, a 90% absorption bandwidth was observed between 4.1–10.6 GHz in the simulation results and between 3.4–10.8 GHz in the measurement results. Moreover, when the SGP was in the OFF state, a 90% absorption bandwidth was observed between 2.3–5 GHz in the simulation results and between 1.7–5.0 GHz in the measurement results. The measured absorption bandwidth was slightly less in the simulation results in both the ON and OFF states than in the measurement results. Table 1 shows a comparison of the proposed metamaterial absorber using an SGP with those of other earlier reports.

**Table 1:** Comparison of the proposed metamaterial absorber using an SGP with those of other earlier reports.

Reference paper	Tuning Method	ON state			OFF state		
		Absorption Band (GHz)	BW^a^ (%)	Microwave spectrum	Absorption Band (GHz)	BW (%)	Microwave spectrum
^35^	PIN diode	2.85–3.15	10	S-	3.8–4.2	10	S-
^36^	PIN diode	12–12.8	6.4	X-	7.1–8.1	13.2	C-
^37^	PIN diode	4.75–4.85	2	C-	4.1–4.2	2.4	C-
^38^	PIN diode	8.4–9.3	10.2	X-	9.2–10.5	13.2	X-
^39^	Liquid Metal	7.43–14.34	63	C-, X-	5.62–7.3	26	C-
Proposed work	PIN diode	4.21–10.46	85	C-, X-	1.7–5.13	100	L-, S-, C-

^a^$$BW={\rm{\Delta }}f/{f}_{c},\,where\,\Delta f={f}_{high}-{f}_{low}\,and\,{f}_{c}=({f}_{high}+{f}_{low})/2$$.

## Methods

### Simulation

To perform our analysis, we used the ANSYS high frequency structure simulator (HFSS). After designing only one unit cell for simulation, we set up the master and slave as two pairs in order to assume an infinite periodic structure. Two different planes (XZ−, YZ−) were assigned as master/slave pairs. One pair of the floquet ports was used as the excitation port. An air box with a size of 11 × 11 × 33 mm^3^ was 13.5 mm away from the top and bottom layers. The conductivity of the copper used was 58 × 10^6^ S/m and its thickness was 35 μm. Because the PIN diode exhibits an inductive/capacitive impedance of 0.7 nH/0.21 pF when in the ON/OFF state, respectively, we set up the ON and OFF states of the PIN diode to 3 Ω and 1 MΩ in the simulation, which made it operate as a short and open circuit, respectively.

### Measurement

To perform our measurements, we set up for normal incidence in free space, as shown in Fig. 6. In order to prevent the influence of signals reflected from objects other than the prototype, we placed wedge-tapered absorbers around the sample. We measured only the reflection coefficient using a Dorado AN-GH1-18N (with a frequency range from 1 to 18 GHz) single horn antenna and an Anritsu MS2038 VNA because the bottom of sample was totally covered with copper. Therefore, there was no transmitted signal. In addition, we used a time-gating method for measuring only the reflected signals. To satisfy the far-field condition, the distance between the sample and the horn antenna was 1 m and a separate DC power supply device was used to activate the PIN diodes. The fabricated absorber sample consisted of 2916 PIN diodes. When all the diodes were turned on, a power of 0.5 W was consumed with a DC voltage of 0.75 V.

## Discussion

In this study, we proposed a broadband frequency-reconfigurable metamaterial absorber using a switchable ground plane (SGP). On the SGP, we mounted PIN diodes and multilayer structures. Based on the ON/OFF state of the PIN diodes of the middle layer (the SGP layer), when the thickness of the unit cell is varied, variations in the absorption frequency value occur, because the thickness of the unit cell affects the reflection coefficient. We have not only proposed a frequency reconfigurable absorber, but also employed multi-resonant and resistive components for broadband frequency absorption. The proposed unit cell consists of two square rings with a chip resistor. The inner ring determines the absorption frequency for lower frequencies and the outer ring determines the absorption frequency for higher frequencies. To theoretically demonstrate the performance of the proposed structure, we performed simulations using a full-wave simulation tool, and fabricated a sample with a size of 297 × 297 mm^2^ with 27 × 27 unit cells. The conductive pattern of the unit cell structure was fabricated using a PCB etching method and the electronic components were mounted via an SMT process. We performed experiments on the fabricated sample using a normal incidence set-up. Additionally, we used a time-gating method for measuring only the reflected signals. As a result, when the SGP was in the ON state, the 90% absorption frequency band according to our simulation results was from 4.21 to 10.46 GHz, and it was from 3.4 to 10.8 GHz according to our measurement results. When the SGP was in the OFF state, the 90% absorption frequency band according to our simulation results was from 1.7 to 5.13 GHz, and it was from 1.7 to 5.0 GHz according to our measurement results. Therefore, the proposed SGP and the frequency-switching capabilities of the broadband metamaterial absorber are functioning successfully. The proposed absorber is useful for practical EM absorber applications, such as EM stealth technology, which requires broad bandwidths.
